# The Arabidopsis Cop9 signalosome subunit 4 (CSN4) is involved in adventitious root formation

**DOI:** 10.1038/s41598-017-00744-1

**Published:** 2017-04-04

**Authors:** Daniel Ioan Pacurar, Monica Lacramioara Pacurar, Abdellah Lakehal, Andrea Mariana Pacurar, Alok Ranjan, Catherine Bellini

**Affiliations:** 1 Umeå Plant Science Centre, Department of Plant Physiology, Umeå University, SE-90187 Umeå, Sweden; 2 University of Agricultural Sciences and Veterinary Medicine, 400372 Cluj Napoca, Romania; 3 Institut National de la Research Agronomic, UMR1318 INRA-AgroParisTech, Institut Jean-Pierre Bourgin, Univ. Paris-Sud, F-78000 Versailles, France; 4 SweTree Technologies AB, P.O. Box 4095, SE-904 03 Umeå, Sweden

## Abstract

The COP9 signalosome (CSN) is an evolutionary conserved multiprotein complex that regulates many aspects of plant development by controlling the activity of CULLIN-RING E3 ubiquitin ligases (CRLs). CRLs ubiquitinate and target for proteasomal degradation a vast number of specific substrate proteins involved in many developmental and physiological processes, including light and hormone signaling and cell division. As a consequence of CSN pleiotropic function, complete loss of CSN activity results in seedling lethality. Therefore, a detailed analysis of CSN physiological functions in adult Arabidopsis plants has been hampered by the early seedling lethality of *csn* null mutants. Here we report the identification and characterization of a viable allele of the Arabidopsis COP9 signalosome subunit 4 (*CSN4*). The allele, designated *csn4-2035*, suppresses the adventitious root (AR) phenotype of the Arabidopsis *superroot2-1* mutant, potentially by altering its auxin signaling. Furthermore, we show that although the *csn4-2035* mutation affects primary and lateral root (LR) formation in the *2035* suppressor mutant, *CSN4* and other subunits of the *COP9* complex seem to differentially control AR and LR development.

## Introduction

The CSN was first discovered in Arabidopsis, during a screening for mutants exhibiting constitutive photomorphogenic development in darkness, and was subsequently shown to be evolutionary conserved across eukaryotes (reviewed in ref. 1). The complex is composed of eight subunits, CSN1-CSN8. Six (CSN1-CSN4, CSN7 and CSN8) contain a PCI (Proteasome, COP9 signalosome and eukaryotic initiation factor 3, eIF3) domain, and two (CSN5 and CSN6) contain a MPN (Mpr1p-Pad1p-N-terminal) domain^2^. In Arabidopsis, the PCI domain-containing subunits are encoded by single copy genes, while the MPN domain-containing subunits are each encoded by two highly homologous genes. The two genes encoding CSN5 (*CSN5A* and *CSN5B*) play unequal roles in the regulation of plant development, while *CSN6A* and *CSN6B*, the genes encoding the CSN6 subunit, act largely redundantly^3^. The PCI and MPN subunits are structurally interdependent during the formation of the COP9 complex, thus explaining why loss of any of the eight CSN subunits leads to an apparently identical seedling lethal phenotype in Arabidopsis^3^. Therefore, a detailed analysis of CSN physiological functions in adult Arabidopsis plants has been hampered by the early seedling lethality of the null *csn* mutants.

Before the availability of T-DNA insertion lines, the only known Arabidopsis *csn* mutants were the pleiotropic seedling lethal mutants, now collectively known as the *cop* (*constitutively photomorphogenic*)/*det* (*de-etiolated*)/*fus* (*fusca*), which were identified through genetic screenings (reviewed in refs 1 and 4). Recent identification of alleles with partial loss of CSN function has shed light on some aspects of CSN functions beyond the seedling stage. However, to our knowledge, viable *csn* mutants are available only for five of the eight CSN subunits, including the double encoded MPN domain-containing subunits CSN5^5^ and CSN6^3^, and only for three out of the six single copy gene-encoded PCI domain-containing subunits, CSN1^6^, CSN2^7^, and CSN3^8^. It has been suggested that one potential reason for the lack of viable known *csn* mutants is that CSN-independent functions of CSN subunits can only be uncovered under specific conditions^4^, and probably in particular types of screening. In this report, we introduce a viable allele of Arabidopsis *CSN4*, identified in a screening for mutants suppressing the adventitious root formation of the auxin overproducer *superroot2* ^9^.

## Results and Discussion

### Isolation of the *2035* mutant and identification of the *csn4-2035* mutation

**Figure 1:**
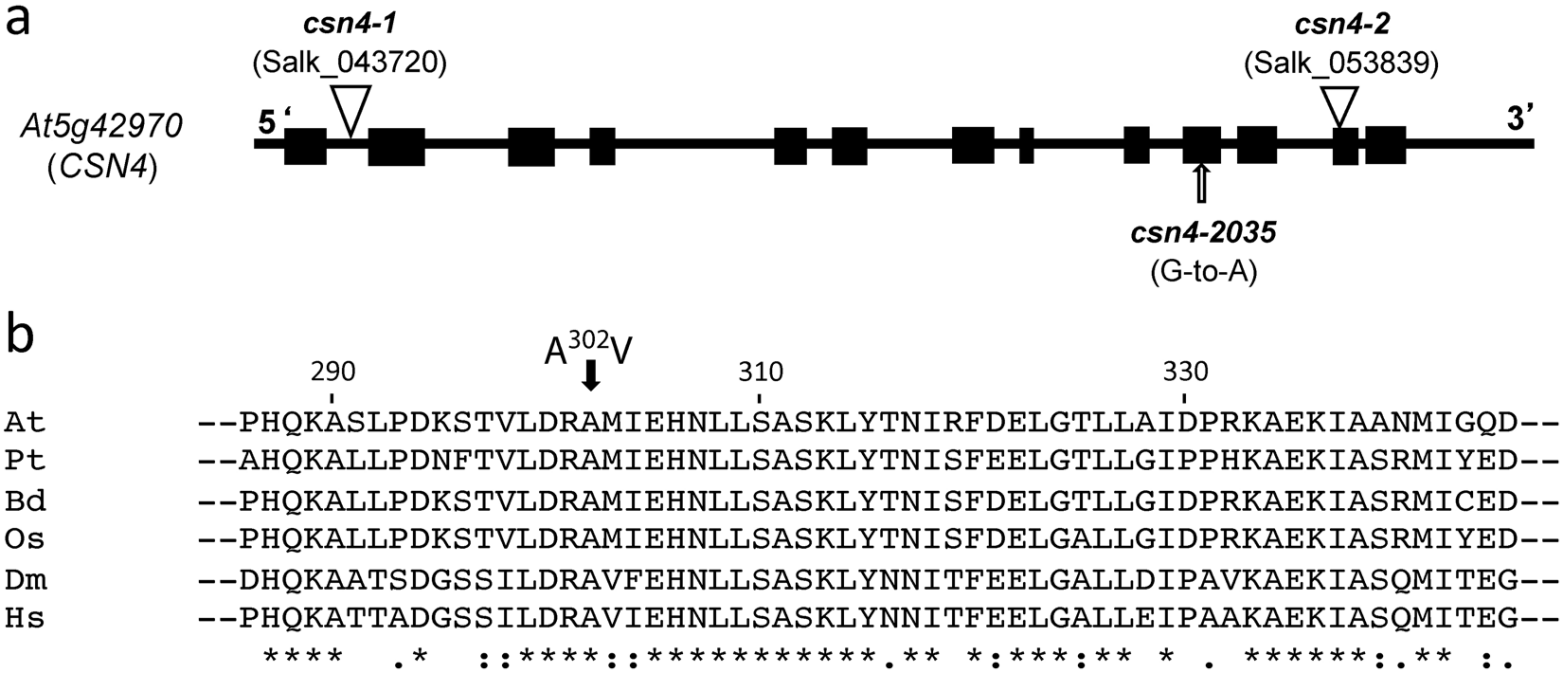
The *csn4* alleles used in this study. **(a)** Structure of the Arabidopsis *CSN4* subunit gene, with the position of the *csn4-2035* point mutation and of the two T-DNA insertion lines. Exons are indicated by black boxes, introns by lines. **(b)** A comparison of a fragment from the Arabidopsis CSN4 protein with homologs from other organisms. The position of highly conserved Ala^302^, mutated in *csn4-2035*, is highlighted. The position and nature of the amino acid substitution found in CSN4-2035 mutant protein is shown. Below, an alignment of CSN4 proteins from *Arabidopsis thaliana* (NP_199111.1; residues 286–345), *Populus tricocharpa* (XP_002320585.1; residues 286–345), *Brachypodium distachyon* (XP_003558584.1; residues 289–348), *Oriza sativa* (NP_001049272.1; residues 289–348), *Drosophila melanogaster* (NP_477444.1; residues 294–353) and *Homo sapiens* (NP_057213.2; residues 287–346), around Arabidopsis Ala^302^ is shown.

Aiming to identify new Arabidopsis genes involved in the control of adventitious root (AR) formation, we screened for suppressors of the *superroot2-1* (*sur2-1*) mutant^10^. We isolated, mapped and characterized a number of suppressors, and, for a subset of mutants, we identified the causal mutations in the corresponding genes^9^. Using the combined advantages of classical map-based-cloning^11^ and whole genome re-sequencing^12^, we identified a point mutation in the locus At5g42970, which encodes the subunit 4 of the COP9 signalosome complex, as the potential causal mutation for the phenotype of one of the *sur2-1* suppressors, designated *2035*. The mutant carries a G-to-A mutation at position 2592 in the tenth exon of the *CSN4*/*COP8*/*FUS4* gene, which results in an Ala-302-to-Val amino acid substitution (Fig. 1a,b). The Ala^302^ is part of a putative helix-loop-helix domain centered around amino acids 294 and 302^13^. A comparison of the CSN4 protein with homologs from other organisms reveals that the Ala^302^ mutated in *2035* is highly conserved even in more divergent proteins (Fig. 1b), being located in the PCI domain of the protein, that has previously been identified to be critical for the stability of the complex^13^, and recently shown to act as the scaffold for CSN4-6-7 interaction in Arabidopsis^2^. More recently, the crystal structure of the human COP9 signalosome has highlighted the important role of the PCI domain CSN4 subunit in sensing the binding of the neddylated Cullin–RINGE3 ubiquitin ligases to CSN, which is subsequently communicated to CSN5 and CSN6 for de-neddylation^14–16^. Therefore, the mutation of the Ala^302^ could induce a destabilization of the CSN and/or affect the de-neddylation process.

**Figure 2:**
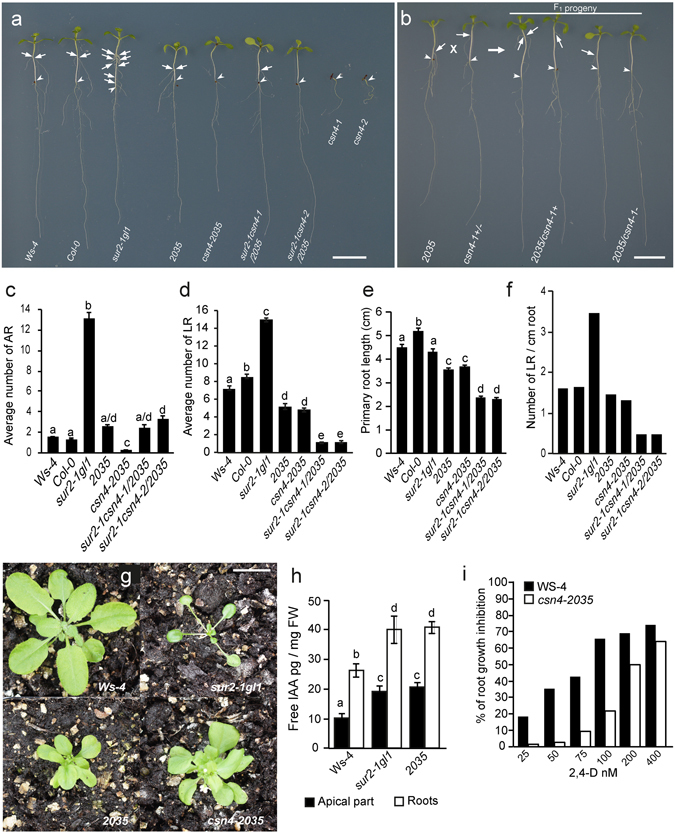
Phenotype and characterization of the *csn4* alleles. **(a)** The phenotype of *in vitro* grown *2035* suppressor mutant, together with *Ws-4*, *Col-0*, *sur2-1gl1*, *csn4-2035*, the two trans-heterozygotes double mutants, *sur2-1csn4-1/csn4-2035* and *sur2-1csn4-2/csn4-2035*, respectively, is shown, as compared to the non-viable *csn4-1* and *csn4-2* alleles. Seedlings were first etiolated in the dark, until their hypocotyls were 6 mm long, and then transferred to light for seven days to induce AR formation on the etiolated hypocotyls. Arrowheads indicate the root-hypocotyl junction; arrows indicate ARs. Bar, 5 mm. (**b)** Allelism test. A cross between the homozygous *2035* and the heterozygote *csn4-1* gives a 1:1 wild-type to mutant phenotype segregation ratio in the F_1_ generation. Arrowheads indicate the root-hypocotyl junction; arrows indicate ARs. Bar, 5 mm. **(c)** Numbers of AR were counted on the hypocotyls treated as in (**a**), and averaged. **(d)** Numbers of emergent lateral roots were counted on seedlings grown *in vitro* directly in light conditions for 10 d and averaged. **(e)** Primary root length was measured on the same seedling as in **(d)** and averaged. **(f)** Lateral root density was estimated by dividing the average number of lateral roots (**d**) by the average root length (**e**). **(g)** Phenotypes of 3-week-old wild-type, *sur2-1gl1*, *2035* and *csn4-2035* mutant plants. Seeds were sown *in vitro* in light conditions and were subsequently transferred to soil where the plants were grown for 3 weeks in a controlled environment, as described in the Methods. Bar, 20 mm. For (**c** to **e**) at least 50 seedlings of each line were analyzed and the experiments were repeated three times and the data pooled. Error bars indicate standard error. One-way ANOVA combined with Tukey’s multiple-comparison post-test revealed that the values indicated by different letters were significantly different from each other (P < 0.05; n > 150). (**h**) Free IAA content was quantified on apical parts (cotyledons+hypocotyls) and on roots of seedlings grown *in vitro* under light for nine days; samples were extracted, purified, and analyzed by GC-MRM-MS as described by^55^. Three biological replicates of at least 50 seedlings from each line were analyzed and averaged. Error bars indicate standard error. One-way ANOVA combined with Tukey’s multiple-comparison post-test revealed that the values indicated by different letters were significantly different from each other (P < 0.05; n = 3). **(i)** csn4-2035 is slightly resistant to exogenously applied auxin. Percentage inhibition was calculated by dividing the average growth on 2,4-D containing medium by the average growth on control medium and subtracting this ratio from 100% (n > 25).

Segregation analysis of the F_2_ progeny from a *sur2-1gl1* × *2035* cross showed a 3:1 ratio of superroot:suppressor phenotype consistent with a single recessive mutation^9^. The mutant is viable and fertile as a homozygote, both in the *sur2-1* mutant and in the *Ws-4* wild-type backgrounds and does not exhibit the characteristic *csn* mutant phenotype, in contrast to seedling lethal *csn4-1* (Salk_043720) and *csn4-2* (Salk_053839) T-DNA insertion alleles, both isolated in the *Col-0* background (Fig. 2a and ref. 17). To demonstrate unambiguously that the mutation in the suppressor *2035* affects *CSN4*, we conducted an allelism test using both insertion alleles. Due to early seedling lethality of the homozygous T-DNA insertion alleles, we crossed the homozygous suppressor *2035* with heterozygote *csn4-1/*+ and *csn4-2/*+ plants. A 1:1 mutant to wild-type phenotype segregation ratio was observed in the F_1_ generation of both crosses, as shown in Fig. 2b for the cross with *csn4-1*. The individuals that were potential *csn4* trans-heterozygote mutants had flat cotyledons, shorter hypocotyls than wild-type like *sur2-1/*+ heterozygote plants, and no AR, except for a few individuals developing only one AR, as compared to *sur2-1/*+ wild-type like plants which on average, developed 1.8 AR/hypocotyl. We genotyped all the F_1_ individuals and confirmed that in the progeny of both combinations of crosses, all seedlings with a short hypocotyl and flat cotyledons were trans-heterozygotes, carrying either *csn4-1*/*csn4*-*2035* or *csn4-2*/*csn4-2035* hetero-allelic combination, while seedlings with a wild-type phenotype did not carry any T-DNA insertion in the *CSN4* gene and were heterozygote for what we called the *csn4-2035* allele. All trans-heterozygous mutants were viable and grew in soil, indicating that the mutation in the suppressor *2035* was responsible for the observed phenotypes.

### Phenotypic characterization of the suppressor *2035*

When grown *in vitro* on vertical plates, *sur2-1gl1* developed numerous AR on the etiolated hypocotyls^9^. The suppressor *2035*, which carries the mutation *csn4-2035* in a *sur2-1gl1* background, developed significantly fewer AR compared to *sur2-1gl1* (Fig. 2a,c). In addition, the AR formation was almost completely abolished in the *csn4-2035* single mutant compared to the *Ws-4* wild-type, since the majority of the seedlings had no initiated AR on the hypocotyl seven days after being transferred to the light, and only a limited number of individuals developed one AR (Fig. 2a,c). We had previously shown that the main root system of suppressor *2035* was also significantly reduced compared to *sur2-1gl1* (Fig. 2d,e and ref. 9). Here we show that the main root system is also reduced in the *csn4-2035* single mutant as compared to the *Ws-4* wild-type. Both the primary root (PR) length and the lateral root (LR) number were significantly reduced (Fig. 2d,e,f), confirming that the *csn4-2035* mutation also affects PR and LR formation.

Interestingly, the AR number *vs* LR number and PR length were differentially affected by the *csn4-1*/*csn4-2035* and *csn4-2*/*csn4-2035* heteroallelic combinations in the two trans-heterozygote double mutants, *sur2-1csn4-1/2035* and *sur2-1csn4-2/2035*. While the average number of AR in the trans-heterozygote double mutants, *sur2-1csn4-1/2035* and *sur2-1csn4-2/2035* was not affected compared to *2035* (Fig. 2c), the PR length and the LR number and density were significantly decreased (Fig. 2d,e,f). As shown in Fig. 2c, the number of AR was not significantly different in the two populations derived from *sur2-1csn4-1/2035* and *sur2-1csn4-2/2035* compared to the *2035* suppressor. This was surprising, since it has been reported that the phenotype of a hypomorph allele had a more severe effect in *trans* to a deletion allele than when homozygous^18^. Indeed, a more extreme phenotype in the populations containing 50% *trans* mutants was observed for PR and LR development, since the LR density was decreased in the two populations to 32% and 34%, respectively as compared to *2035* (Fig. 2d,e,f). It should be noted that in Fig. 2c–f, the data corresponding to the trans-heterozygotes *sur2-1csn4-1/2035* and *sur2-1csn4-2/2035* were collected from segregating populations including viable *sur2-1csn4-1/2035* (50%) and *2035* (25%), and *sur2-1csn4-2/2035* (50%) and *2035* (25%), respectively. These were generated by self-pollinating the *sur2-1csn4-1/2035* and *sur2-1csn4-2/2035* parents, respectively. In both situations, the segregating *sur2-1csn4-1* −/− (25%) and *sur2-1csn4-2* −/− (25%) had the characteristic *fusca* phenotype and were not considered for the characterization. Apparently, losing one copy of the *csn4-2035* in 50% of the individuals from both populations did not have an impact on the AR number compared to the suppressor *2035*, but this lack could not be compensated for in the case of PR and LR formation. This suggests that *CSN4* and/or the *COP9* complex differentially control AR and LR development.

When grown in soil, in LD conditions, both *2035* and *csn4-2035* develop smaller rosettes than the wild type (Fig. 2g), and the adult plants show a mild dwarfism and loss of apical dominance phenotype as observed for other hypomorphic *csn* mutants^7^.

The seedling and rosette phenotype of *2035* (short petioles, round leaves) is the result of the suppression of the *sur2-1* phenotype. The apical part (not only the roots) loses the auxin related phenotype. Since the *sur2-1* mutant phenotype is the result of auxin overproduction, its suppression may be due to either altered auxin homeostasis or auxin perception in the suppressor mutant. It has been reported that the viable *CSN* alleles *csn1-10*, *csn2-5*, *csn3-3*, *csn5a* and *csn5b* have altered auxin perception and auxin-resistant root growth^5–8, 19^. To test whether the *csn4-2035* mutation suppresses the *sur2-1* phenotype by disturbing the auxin signaling or the auxin homeostasis in the *2035* mutant, we measured the free IAA content both in the apical parts and the roots of *in vitro* grown seedlings. As we previously described in ref. 9 and show here in Fig. 2h, the *2035* mutant retains the high auxin level of *sur2-1gl1*, which indicates that the reduction of the AR number in *2035* is unlikely to be due to a reduced auxin content, suggesting a possible alteration in auxin signaling.

### Auxin signaling is perturbed in *2035*

It is well-known that the CSN plays a role in auxin signaling, acting as a de-neddylase to regulate SCFTIR1/AFB activity, and mutants altered in any CSN subunit are more or less resistant to inhibition of root growth by exogenously applied auxin and have reduced lateral root formation^5–7, 20^. Therefore, we performed a dose-response assay and measured the auxin inhibition of root elongation and found that *csn4-2035* was resistant to 2,4-D compared to Ws-4 (Fig. 2i and Supplementary Fig. S1). After transfer to the medium supplemented with 0.1 μM 2,4-D, the wild type *Ws-4* displayed inhibition of root elongation by 65% whereas *csn4-2035* seedlings displayed a 20% inhibition only (Fig. 2i) and did not develop LR (Supplementary Fig. S1).

**Figure 3:**
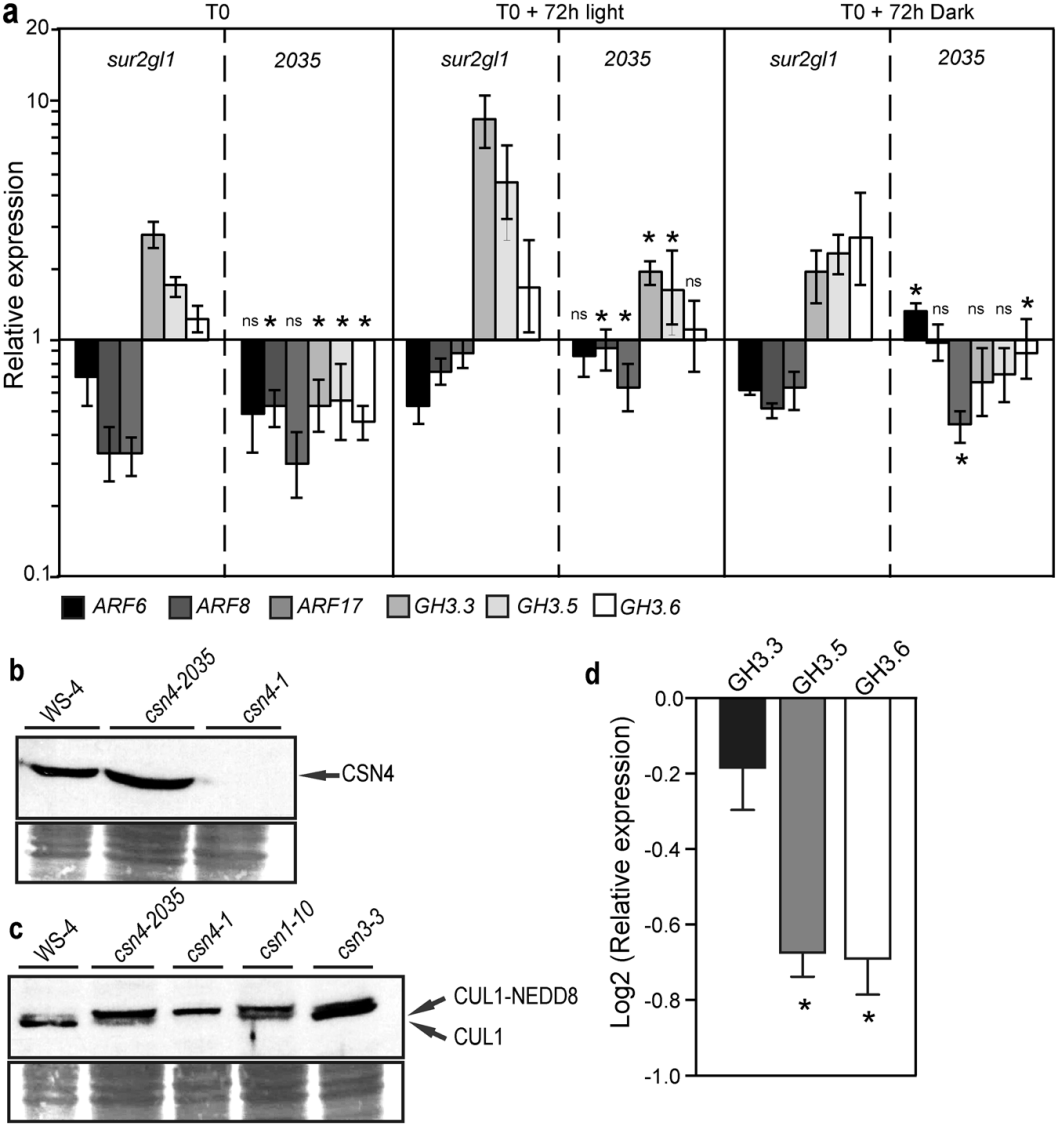
Auxin signaling is perturbed in *2035*. **(a)** Quantification by quantitative real-time PCR of *ARF6*, *ARF8*, *ARF17*, *GH3.3*, *GH3.5* and *GH3.6* transcript abundance in hypocotyls of *sur2-1gl1* and *2035* seedlings, which were etiolated until their hypocotyl had reached 6 mm (T0) and then transferred to the light for 72 h (T0+72 h light), or kept in dark for an additional 72 h (T0+72 h dark). Gene expression values are relative to the expression in the wild type, for which the value is set to 1. Error bars indicate standard error obtained from three independent biological replicates; (*) indicates values that were significantly different from *sur2-1gl1* values according to one-way ANOVA combined with Bonferroni’s comparison post-test; (ns) indicates values not significantly different; (*P* < 0.05; *n* = 3). **(b)** *csn4-2035* is a weak allele mutant that produces as much protein as the wild type. CSN4 western blot analysis of protein extracts prepared from WS-4, *csn4-2035* and the null allele *csn4-1*. **(c)** *csn4-2035* mutation affects the de-neddylation activity of CSN4 protein. CUL1 western blot analysis of protein extracts prepared from Ws-4 and *csn* mutant seedlings. The upper band indicates the modified (neddylated) CUL1. Ponceau stained polyvinylidene difluoride (PVDF) membrane is shown as a loading control. (**d**) Quantification by quantitative real-time PCR of GH3.3, GH3.5 and GH3.6 transcript abundance in hypocotyls of csn4-2035 seedlings. Gene expression values are relative to the expression in the wild type, for which the value is set to 1. Error bars indicate standard error obtained from three independent replicates; (*) indicate values that were significantly different from wild-type values (P < 0.05; n = 3).

In previous work, we have demonstrated that a regulatory module, composed of three *AUXIN RESPONSE FACTOR* genes (*ARF6*, *ARF8*, and *ARF17*) and three auxin-responsive genes (*GH3.3*, *GH3.5*, and *GH3.6*) controls AR initiation in Arabidopsis hypocotyls by modulating JA homeostasis^21, 22^. In addition, we recently showed that selected suppressor mutations of the *sur2-1* phenotype differentially affected the expression of the ARF/GH3 regulatory module^9^. In the suppressors analyzed, the reduced number of AR was independent of the endogenous auxin content, but positively correlated with the transcript amount of the three *GH3* genes^9^. In the present work we show that despite the endogenous content of free IAA in the suppressor *2035*, which is still as high as that of the *sur2-1gl1* mutant (Fig. 2h and ref. 9), the relative transcript amount of the *GH3* genes is significantly reduced compared to *sur2-1gl1* (Fig. 3a), probably explaining the reduced number of AR. Interestingly, this could be explained neither by a down-regulation of the expression of the positive regulators *ARF6* or/and *ARF8* nor an up-regulation of the negative regulator ARF17 (Fig. 3a). ARF transcriptional activity is negatively regulated by transcriptional repressors of the Aux/IAA family, which are unstable proteins rapidly degraded through the SCF^TIR1/AFB1-3^ dependent ubiquitin-proteasome system in the presence of a high auxin concentration. It was also shown that mutants altered in the expression of CSN3, CSN4, CSN5 or CSN8 subunits were partially impaired in the SCF^TIR1/AFB1-3^-mediated protein degradation, resulting in a partially altered auxin response^5, 17^.

Because the CSN cleaves the RUB/NEDD8 peptide from the cullin subunit of CRL ubiquitin-ligases, we checked whether or not the *csn4-2035* mutation affected CUL1 modification. We first showed that the *csn4-2035* mutant produced as much CSN4 protein as the wild type, while the null allele mutant retained no protein as expected (Fig. 3b). These results suggested that the phenotype was not the result of a reduced amount of CSN4 protein but most likely due to a modification of its activity or to a defect in the CSN assembly or structure. Therefore, we analyzed de-neddylation activity (Fig. 3c). We included *csn1-10* and *csn3-3* mutants as controls because they were shown to exhibit similar auxin resistance but had different de-neddylation activity^8^. Figure 3c shows that there was no de-neddylation of CUL1-NEDD8 in the *csn4-1* null allele mutant as expected and a clear accumulation of de-neddylated CUL1 in the wild type and *csn3-3* mutant as reported in previous studies^8^. In contrast, we observed an accumulation of CUL1-NEDD8 in *csn4-2035*, as is the case in *csn10-1*, indicating that the *csn4-2035* mutation partially affects the de-neddylation activity of the CSN and, as a consequence, decreases auxin sensitivity. This hypothesis is supported by the slight but significant down-regulation of the auxin inducible *GH3* genes in the *csn4-2035* mutant (Fig. 3d).

Therefore, the down-regulation of the auxin inducible *GH3* genes in the *2035* suppressor mutant, resulting in the reduced number of AR, can reasonably be explained by an inefficient degradation of AUX/IAA proteins and, as a consequence, the down-regulation of the auxin signaling pathway which in turn would induce the expression of the activating ARFs such as *ARF6* and *ARF8* in a regulatory feedback loop. This would explain why, despite high endogenous auxin content and a combined increase in the relative transcript amount of the *ARF6* and *ARF8* positive regulators and decrease in the transcript amount of the *ARF17* negative regulator, compared to *sur2-1gl1* (Fig. 3a), the mutant *2035* develops fewer AR.

### *csn4*-*2035* mutation affects blue and red light perception in a *sur2*-*1* background

No obvious *csn* phenotypes - *cop/det* (open cotyledons, short hypocotyl and absence of apical hook of the dark-grown seedling) and *fusca* (accumulation of anthocyanins) - typical of the other reported Arabidopsis mutants of all six PCI-domain subunits, including the two *CSN4* T-DNA alleles *csn4-1* and *csn4-2*, were observed, either in *2035* mutant or in the *csn4-2035* single mutant (Fig. 2a). Grown *in vitro*, in LD conditions, the hypocotyl of *2035* had an intermediate length between that of the wild-types and *sur2-1gl1* ^9^. Early observations have shown that light-grown *CSN* null mutants have very short hypocotyls^23^. Shorter hypocotyls than the wild-type controls have also been observed in week *CSN* alleles^5, 19^, and the role of CSN in hypocotyl elongation has recently been demonstrated^24^. In a screening for novel CSN interactors, the authors identified CFK1 (COP9 INTERACTING F-BOX KELCH 1), a new plant-specific CSN-interacting F-box protein that is regulated by the CSN and the proteasome-dependent proteolysis. This physical interaction suggests that CFK1 function might be required for CSN-mediated hypocotyl inhibition, as demonstrated by enhanced hypocotyl length in the double mutant *csn5a-2 CFKRNAi* compared to either parents^24^. The authors have shown that light induces accumulation of the CFK1 transcript in the hypocotyl, and that CFK1 promotes hypocotyl elongation by increasing cell size. Moreover, while reduction of CSN levels enhances the short hypocotyl phenotype of *CFK1RNAi* seedlings, complete loss of CSN activity suppresses the long-hypocotyl phenotype of CFK1-overexpressing seedlings. In light of these findings, we can speculate that suppression of the hypocotyl growth in the *2035* suppressor mutant compared to *sur2-1gl1* could be explained by a reduced activity of the *CSN* due to the mutation in the CSN4 subunit.

**Figure 4:**
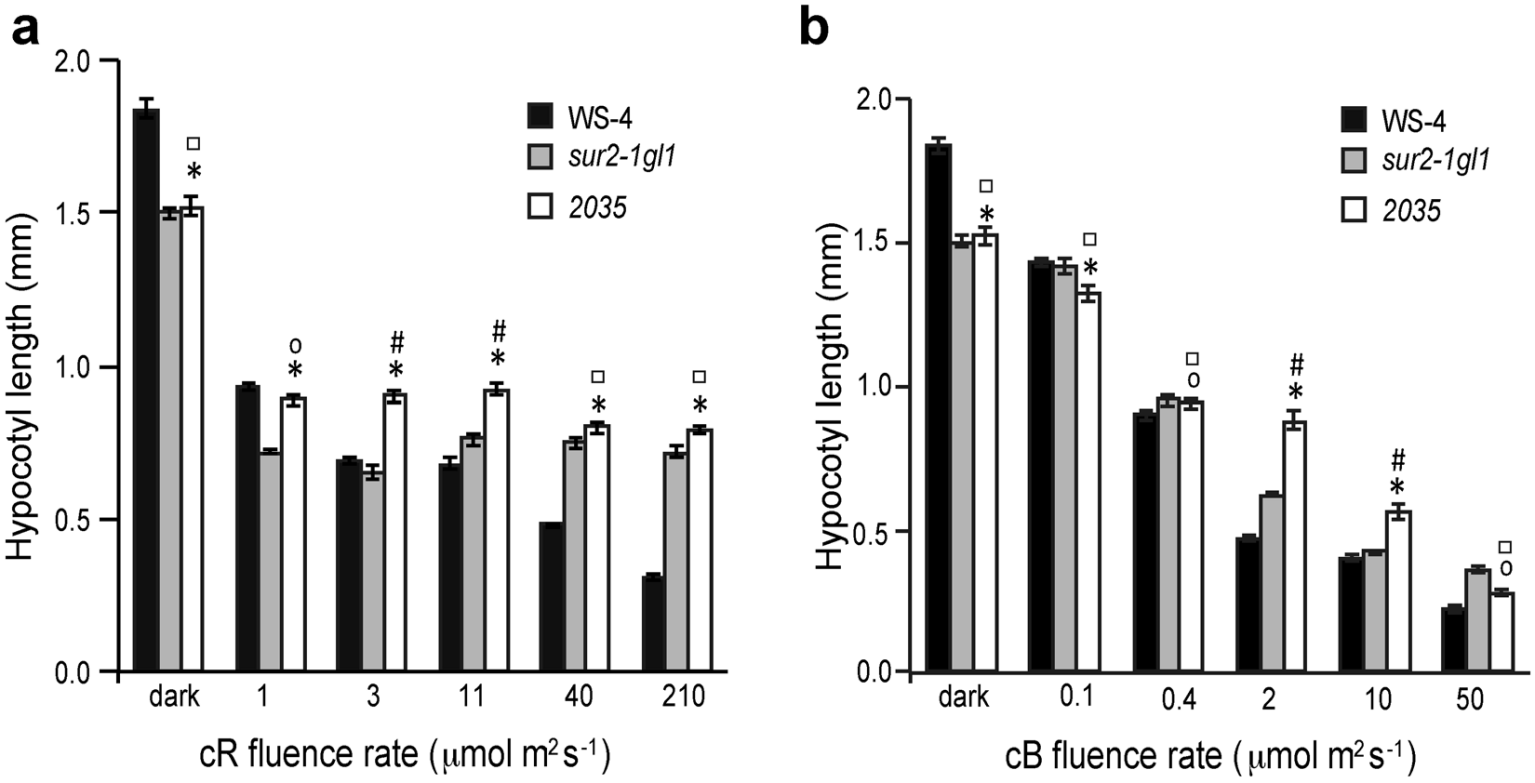
The *csn4-2035* mutation affects red and blue light perception in a *sur2-1gl1* background. All measurements were performed on three independent biological replicates with a minimum of 40 seedlings each, as described in the Methods. Error bars indicate standard error. One-way ANOVA combined with Tukey’s multiple-comparison post-test revealed that the values indicated by (*) were significantly different from *Ws-4* values; (#) indicates values significantly different from *sur2-1gl1* values; while (o) and (◻) indicate values not significantly different from *Ws-4* and *sur2-1gl1* values, respectively; (P < 0.05; n > 120).

In Arabidopsis, normal hypocotyl elongation is controlled both by cryptochromes, which respond to blue/UV-A light, and phytochromes, which sense red/far-red light. An allelic mutant of *sur2-1*, *red1* (*r* *ed* *e* *longate* *d1*), has been identified during a screening for mutants that display enhanced etiolation in continuous red light (cR), suggesting a defect in red light perception^25^. In addition, the CSN was originally discovered as an essential complex that regulates light-induced development in Arabidopsis^26–29^, and increased photomorphogenic responses were observed in different light conditions for viable alleles of the CSN subunit genes, *csn5a*, *csn5b* ^5^ and *csn6a-1* and *csn6a-2*, respectively^3^. To investigate further whether the *csn4-2035* mutation alters light perception, we measured the hypocotyl length of *2035*, *sur2-1gl1* and *Ws-4* under five fluence rates of continuous red (cR) and continuous blue (cB) light. In the dark, *2035* and *sur2-1gl1* had the same hypocotyl length and were shorter than the wild type, as previously described for *sur2-1* (Fig. 4a,b and ref. 10). When grown under cR at 40 or 210 μmol m^−2^ s^−1^ irradiance, both *sur2-1gl1* and *2035* were longer than the wild type. Nevertheless the *2035* mutant had a significantly longer hypocotyl compared to *sur2-1gl1* when grown under 1, 3 or 11 μmol m^−2^ s^−1^ irradiance (Fig. 4a), suggesting that a mutation in the CSN4 subunit affects red light perception. Similarly, a significant difference between the suppressor mutant *2035* and *sur2-1gl1* was observed when seedlings were grown under cB light at 2 and 10 μmol m^−2^ s^−1^ irradiance (Fig. 4b). From these data, we can conclude that a mutation in the CSN4 subunit affects red and blue light perception to some extent, but we cannot say whether the AR phenotype and the light related phenotype are connected, and additional experiments are required. Nevertheless, we have previously shown that AR initiation is also controlled by light^21, 30^, as well as by a signaling cross-talk between auxin and jasmonate involving MYC2 transcription factor^22^, therefore the *csn4-2035* mutation could also highlight cross-talk between light and these signaling pathways in the control of AR initiation. Indeed, it was recently suggested that inhibition of hypocotyl elongation by jasmonates is enhanced under red light in phyB-dependent manner^31^, and that JA-related transcription factors MYC2, MYC3, and MYC4 are short-lived proteins degraded by the proteasome, and stabilized by JA and light, in *Arabidopsis thaliana*. Darkness and CONSTITUTIVE PHOTOMORPHOGENIC1 (COP1) destabilize MYC2, MYC3, and MYC4 proteins, whereas red and blue lights stabilize them through the activation of the corresponding photoreceptors^32^. Therefore, the *csn4-2035* mutation, by perturbing red and blue light perception, may also affect the stabilization of MYC proteins and thereby have an impact on adventitious root formation.

### CSN subunits play differential roles in AR and LR formation

The CSN regulates multiple plant hormone signaling and developmental processes through SCF-type CRLs, known as SCF complexes, which act in many pathways such as SCF^TIR1^ in auxin^7, 20^, SCF^COI1^ in jasmonate^33, 34^ and SCF^SLY1^ in gibberellic acid signaling^35^, SCF^*UFO*^ in flower development^36^, and SCF^CFK1^ in hypocotyl elongation^24^. The CSN also controls photomorphogenic development and abscisic acid signaling through a CUL4-CDD (COP10, DDB1 and DET1^37^), and a CRL4-DDA1 complex^38^, respectively. The isolation of viable *csn* mutants provides an opportunity to study CSN functions beyond seedling stages by elucidating the role CSN and its individual subunits play in these processes. Interestingly, the viable *CSN* mutant alleles described so far, both for the double encoded MPN subunits and single encoded PCI subunits, either display pleiotropic developmental defects or have subtler phenotypes. The first viable *csn* mutants described were the loss-of-function T-DNA insertion mutants in the double encoded MPN domain-containing subunit *CSN5A* and *CSN5B*, *csn5a-1* (null), *csn5a-2* (hypomorphic), and *csn5b-1* ^5^. The *csn5a* mutant alleles display increased photomorphogenic responses and pleiotropic developmental defects both at the seedling and adult stage, affecting lateral root and root hair formation, and flower size. In contrast, the *csn5b* mutant shows comparably subtle phenotypes^5^. However, both *csn5a-1csn5-b* and *csn5a-2csn5b* double mutants mimic the phenotype of the previously described *cop/det/fus* mutants, indicating that the two *CSN5* genes have redundant functions, with *CSN5A* having a stronger relative contribution to the respective phenotype than *CSN5B*. On the other hand, except for a very mild photomorphogenic phenotype in the dark and under blue light, the T-DNA insertion mutants in the *CSN6A* and *CSN6B* genes, coding for the second MPN subunit, do not display any obvious morphological defects in white light and after the seedling stage, while loss of function for both CSN6 proteins leads to seedling lethality and a *fusca* phenotype in the double mutant *csn6a-1csn6b-1* ^3^.

Of the three single gene encoded PCI subunits for which viable alleles have been identified, *csn1-10* has been described to have severe pleiotropic developmental defects associated with altered auxin responses^6^, while *csn2-5*, except for a mild dwarfism of adult plants^7^ and a “curly hypocotyl” phenotype when seedlings were grown in darkness^19^ and *csn3-3* ^8^, although impaired in auxin responses, retain a wild-type like phenotype, under the conditions investigated and for the analyzed parameters.

**Figure 5:**
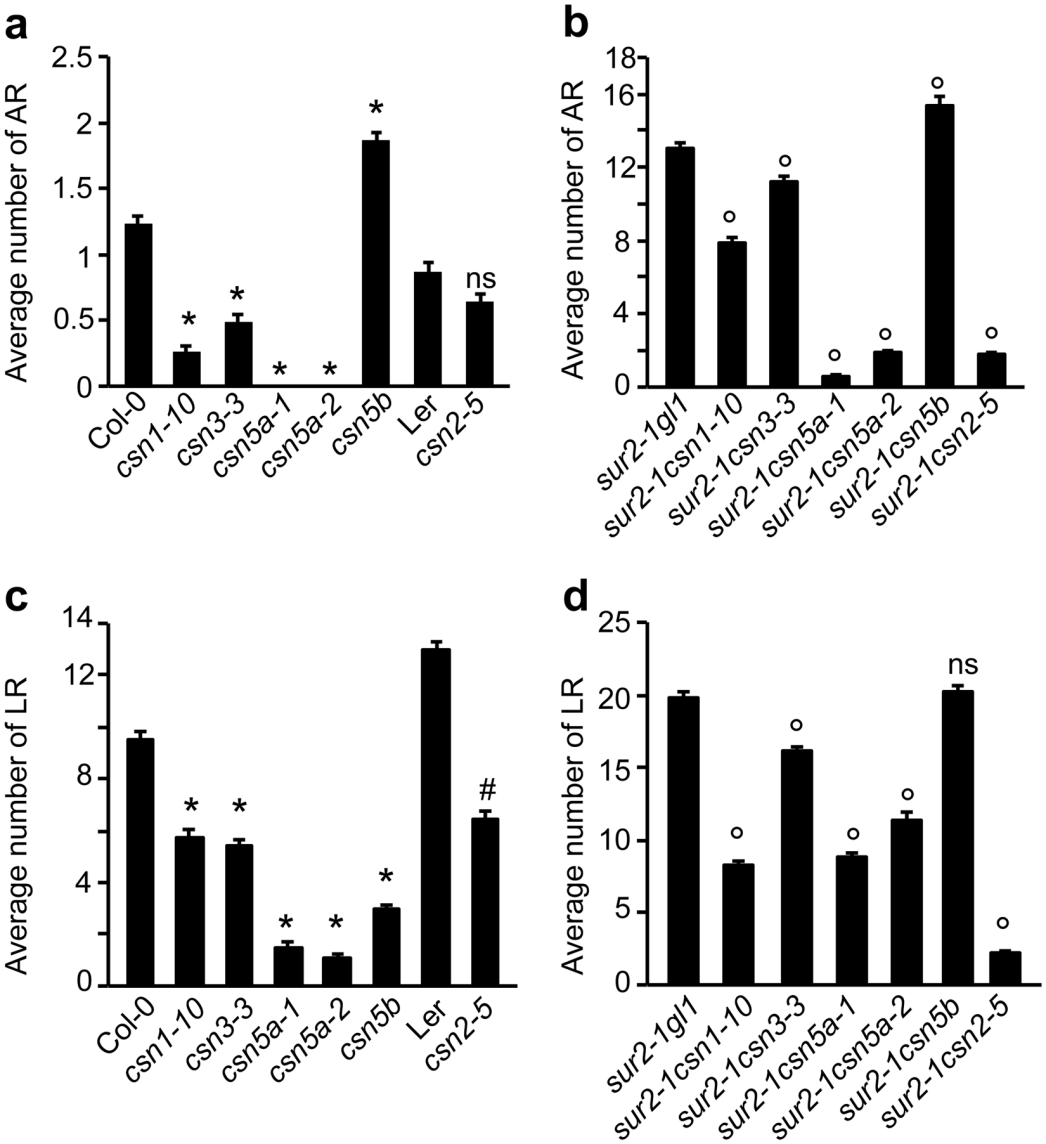
CSN subunits play differential roles in AR and LR formation. **(a,b)** Adventitious roots were counted on seedlings first etiolated in the dark, until their hypocotyls were 6 mm long, then transferred to the light for 7 d, and averaged. **(c,d)** Numbers of emergent lateral roots were counted on seedlings grown *in vitro* directly in light conditions for 10 d and averaged as described in the Methods. At least 30 seedlings of each line were analyzed, the experiments were repeated three times and the data pooled. Error bars indicate standard error; A one-way ANOVA combined with Dunnett’s comparison post-test was used to compare single mutant lines with their respective wild type, and double mutant lines with *sur2-1gl1*; (*) indicates values significantly different from *Col-0* values; (#) indicates values significantly different from L*er* values; (o) indicate values that were significantly different from *sur2-1gl1* values; ns indicates values not significantly different from L*er* (**c**) or *sur2-1gl1* (**d**) values (P < 0.05; *n* > *90*).

In order to test whether the viable *csn* mutants show differences in AR formation and whether they differentially interact with *sur2-1*, we counted the AR number in the single mutants *csn1-10*, *csn2-5*, *csn3-3*, *csn5a-1*, *csn5a-2*, *csn5b* and in the double mutants with *sur2-1*. As shown in Fig. 5a, the AR number varied considerably among the tested *csn* mutants. The *csn1-10* and *csn3-3* mutants developed significantly fewer AR as compared to Col-0, and *csn5a-1* and *csn5a-2* did not form any AR, while *csn2-5* was not significantly different from its corresponding wild-type L*er* (Fig. 5a). Interestingly, in contrast to *csn5a-1* and *csn5a-2*, the *csn5b* mutant developed an increased number of AR compared to the wild-type Col-0 (Fig. 5a), and although all *csn* mutants suppress the AR phenotype of *sur2-1* to various degrees, *csn5b* enhanced the *sur2-1* phenotype (Fig. 5b). This indicates that the two CSN5 subunits differentially contribute to the regulation of AR formation.

As shown in Fig. 2c,d, the *csn4-2035* mutation differentially impacted on AR *vs*. LR development. We wondered whether this was also true for mutations in other CSN subunits. We counted the average number of LR on the seedlings of the *csn1-10*, *csn2-5*, *csn3-3*, *csn5a-1*, *csn5a-2* and *csn5b* single mutants, and in the double mutants with *sur2-1* grown for 10 days under light (Fig. 5c,d). Interestingly, all single and double mutants (except *sur2-1csn5b* double mutant) showed a reduced number of LR compared to the wild-type controls (Fig. 5c) or *sur2-1* (Fig. 5d). Interestingly the number of LR in the *sur2-1csn5b* double mutant was not significantly different from *sur2-1gl1*. Analyzing the impact that the individual *csn* mutations have on AR *vs*. LR development, we can see that the number of both AR and LR is significantly reduced in *csn1-10*, *csn3-3*, *csn5a-1*, *csn5a-2* single mutants compared to Col-0, as well as in their double mutants with *sur2-1*, compared to *sur2-1gl1*. In contrast, the *csn2-5* mutant is affected with respect to LR number, but shows no significant difference in AR number compared to the L*er* wild type, although the *csn2-5* mutation strongly suppresses the *sur2-1* phenotype in the double mutant *sur2*-*1csn2*-*5* (Fig. 5a). The *csn5b* mutant, although showing a significant reduction of the number of LR, developed significantly more AR on the hypocotyl compared to Col-0. Moreover, it enhanced the AR phenotype in the double mutant *sur2-1csn5b*, while the LR number was not significantly different from *sur2-1gl1*.

Mutations in CSN subunits have previously been shown to impact LR formation in the viable identified single *csn* mutants or generated double mutants. While LR numbers in the single mutants *csn1-10* and *csn3-3* ^8^ were comparable to wild-type control, single *csn5a-2*, *csn5b-1* ^5^, as well as double mutants *csn3-3 csn1-10* ^8^, developed significantly fewer LR. This reduction was attributed to the impaired auxin response of the mutants^5, 8^, or to a defect in the cell cycle progression^39^. Nevertheless we have previously shown that jasmonate, through the CORONATIN INSENSITIVE 1 (COI1) signaling pathway, negatively regulates AR initiation in Arabidopsis^22^ while others have shown that it promotes LR formation^40, 41^. Therefore, considering the fact that the COP9 signalosome modulates JA responses by physically interacting with the SCF^COI1^ complex on one hand^33^ and that it modulates JA biosynthesis on the other hand^41^, we can speculate that the different CSN subunits may differentially control AR and LR formation by differentially modulating the JA signaling pathway or JA biosynthesis.

Alternatively, several lines of evidence suggest that light-auxin cross-talk takes place during AR development in Arabidopsis. First, in the *ago1* mutant, the defect in AR formation correlates with an alteration of auxin homeostasis and a hypersensitivity to light^30^. Second, a model for genetic control of AR formation in Arabidopsis hypocotyls that integrates light and auxin signaling has been published^21^. In addition, previous studies suggest that shoot-localized phytochromes regulate lateral root development^42^, and root-localized phytochromes and cryptochromes regulate phototropic responses and growth of primary roots^43–45^. Also, it has been shown that light regulates developmental processes at least in part through cross-talk with the phytohormone auxin^45^. Light signaling involves nucleo-cytoplasmic partitioning of phytochromes^46^ and negative regulators such as CONSTITUTIVE PHOTOMORPHOGENIC 1 (COP1), an E3 ubiquitin ligase involved in 26S proteasome-mediated protein degradation^47^. In darkness, COP1 accumulates in the nucleus to degrade transcription factors including HY5, HYH, HFR and LAF, therefore, suppressing the expression of light-regulated genes^48–50^. On the other hand, light triggers the degradation of COP1 in the nucleus, thereby, activating the expression of light-regulated genes, and promoting photomorphogenesis. The cytoplasmic-nuclear partitioning of COP1 is regulated by the multisubunit COP9 complex (COP9 signalosome or CSN). Total loss-of-function mutations in any CSN components exclude COP1 from nuclear accumulation in the dark^29^ and lead to the extreme seedling-lethal phenotype, whereas partial loss of function mutations in the same subunits highlight differential roles in the regulation of light perception. Therefore, the differential role of the CSN in the regulation of AR and LR formation could also be due to differential regulation of light/auxin signaling cross-talk.

Based on these data, we cannot say whether suppression of the AR phenotype in the *2035* mutant is due to defects in auxin signaling, jasmonate signaling, light perception or light regulatory pathways, cell cycle defects in roots, or a combination of these. However, given the documented function that the COP9 signalosome has in light, auxin and jasmonate signaling, the *csn4-2035* allele could serve for the elucidation of auxin-jasmonate-light cross-talk in AR development. For more general interest, it will provide a tool for a further in-depth study of CSN-independent functions of the CSN4 subunit in Arabidopsis.

## Methods

### Plant material and growth conditions

The *2035* mutant was identified in a previously described screening^9^. In order to avoid selecting wild-type seedlings due to potential contamination of the mutagenized population with wild-type seeds, the *glabra1* mutation was introgressed in the *sur2-1* mutant background. The *glabra1* mutant was identified in the Versailles collection of T-DNA insertion lines and was therefore in the same genetic background as *sur2-1*. Homozygote seeds from the double mutant *superroot2-1gl1* (*sur2-1gl1*) (ecotype Wassilewskija, Ws-4) were mutagenized with ethyl methanesulphonate, as described in ref. 51.

The *csn4-1* (SALK_043720) and *csn4-2* (Salk_053839) segregating lines were described in ref. 17; *csn5a-1* (SALK_063436), *csn5a-2* (SALK_027705) and *csn5b-1* (SALK_007134) were described in ref. 5; *csn2-5* was described in ref. 7; *csn1-10* was described in ref. 6, and *csn3-3* was described in ref. 8. The *2035* mutant is in Wassilewskija (Ws-4) background, the *csn2-5* mutant is in a Landsberg *erecta* (L*er*) background while the rest are in a Columbia (Col-0) background. *In vitro* characterization and auxin quantification were conducted as described previously^9^. For the phenotypic evaluation of soil-grown plants, the seeds were first germinated *in vitro* and subsequently the seedlings transferred into pots. The plants were then placed in growth chambers in long day (16 h light/8 h darkness) conditions, at 22 °C/18 °C (light/dark), 130 μE m^−2^ s^−1^ irradiance on average and 60% relative humidity.

For root growth assays, 6-day-old seedlings grown in the light as described in ref. 30 were transferred to the same medium supplemented with various concentrations of 2,4-D, and root growth was measured after an additional 5 days. The conditions in the controlled environment chambers were as follows: 130 μE m^−2^ s^−1^ irradiance on average, 16/8 light/dark cycle, 22/15 °C, 60% relative humidity. Plates were scanned before and after transfer of the seedling on auxin containing medium. Root length was measured using the ImageJ software package (http://rsb.info.nih.gov/ij/index.html) and ref. 52. Percentage inhibition was calculated by dividing the average growth on 2,4-D containing medium by the average growth on control medium and subtracting this ratio from 100% (n > 25).

### Complementation test and genotyping the *csn4* alleles

For complementation analyses, homozygous *2035* plants were crossed with heterozygous *csn4-1* and *csn4-2*, respectively. F1 progeny was phenotyped and subsequently genotyped, using allele specific PCR primers, as follows. The *csn4-1* mutation was genotyped using CSN4-FW1 and CSN4-RV1.1 to test for the presence of the wild-type gene, and LBb1.3 and CSN4-RV1 to test for the presence of the T-DNA. The *csn4-2* mutation was genotyped using CSN4-FW2 and CSN4-RV2 to test for the presence of the wild-type gene and LBb1.3 and CSN4-RV2 to test for the presence of the T-DNA. To genotype the *csn4-3* point mutation, newly derived cleaved-amplified polymorphic sequence primers were designed using the dCAPS Finder 2.0 software (ref. 53, http://helix.wustl.edu/dcaps/dcaps.html). Two mismatches (underlined) were introduced in the F primer to incorporate a restriction site in the PCR product of one allele. After amplification, the PCR products were digested with HpaI (Fermentas Fast Digest) following the manufacturer’s recommendations and separated on a 4% agarose gel. The *csn4-3* allele yielded two fragments of 150 and 19 bp, while the wild type gave one band of 169 bp. All primers are listed in Supplementary Table S1.

### Hypocotyl growth under different light conditions

Seeds were surface sterilized, sown in rows *in vitro* on media without sugar, stratified for 48 h at 4 °C, then transferred to a plant growth chamber under white light conditions for 4 h to activate germination. Subsequently, five Petri dishes of each light condition/replicate were placed on top of each other, with one chromatography paper layer between them, and wrapped with black plastic leaving the top uncovered (see Supplementary Fig. S2). They were transferred to plant growth cabinets at 20°C with constant blue (cB) or constant red (cR) light, respectively. The light intensity measured on each layer, bottom to top, was as follows: 1; 3; 11; 41 and 210 μmol m^−2^ s^−1^ irradiance of cR light, and 0.1; 0.4; 2; 10 and 52 μmol m^−2^ s^−1^ irradiance of cB light, respectively. For dark growth conditions, five plates/chamber/replicate were wrapped in three layers of aluminum foil and placed vertically. After 7 d, the seedlings were bended on the surface of the medium, the plates were photographed and hypocotyls measured using ImageJ software^52^ (http://rsb.info.nih.gov/ij/index.html). All measurements were performed on three independent biological replicates with a minimum of 40 seedlings each.

### Real-time PCR experiments and data analysis

Transcript abundance was assessed by quantitative real-time (RT) PCR as previously described^22^. All quantifications were repeated with three independent biological replicates, using the following standard thermal profile: 10 min at 95 °C, followed by 40 cycles at 95 °C for 10 s, 60 °C for 15 s (except for *GH3.5* for which the annealing temperature was 65 °C), and 72 °C for 15 s. The sequences of primers used are presented in Supplementary Table S2. Using the RefFinder: http://www.leonxie.com/referencegene.php, *EF1A* (At5g60390) has been validated as the most stably expressed gene, of the four tested, and was used to normalize the RT-PCR data. The expression levels were calculated as previously described^9^. Gene expression values are relative to the expression in the wild type, for which the value is set to 1. All RT-PCR results presented are means from three independent biological replicates. For each independent biological replicate, the relative transcript amount was calculated as the mean of three technical replicates, using the method for calculation of standard errors in relative quantification recommended by^54^.

### Immunoblot analysis

Total proteins were extracted from 10-day-old light grown seedlings in protein extraction buffer (50 mM Tris-HCl pH 7.5, 150 mM NaCl, 0.5% NP40, 1 mM DTT, 1 mM phenylmethylsulfonyl fluoride (PMSF), and Protease Inhibitor cocktail tablets (Roche)^8^. A aliquote of 40 μg of total protein for each genotype was loaded and separated by 10% SDS-PAGE gel. Samples were then blotted and used for immunodetection. anti-CSN4 (1:1000) (COP9 signalosome subunits polyclonal antibody) and anti-CUL1 (1:1000) (CUL1 (*Arabidopsis thaliana*) polyclonal antibody) were purchased at Enzo Life Sciences (http://www.enzolifesciences.com). Goat anti-Rabbit IgG (1:20000) (Agrisera http://www.agrisera.com/en/info/about-agrisera-.html) was used as a secondary antibody.

## Electronic supplementary material

Supplementary information

## References

[1] Wei N, Deng XW (2003). The COP9 signalosome. Annu. Rev. Cell Dev. Biol..

[2] Kotiguda GG (2012). The organization of a CSN5-containing subcomplex of the COP9 signalosome. J. Biol. Chem..

[3] Gusmaroli G, Figueroa P, Serino G, Deng XW (2007). Role of the MPN subunits in COP9 signalosome assembly and activity, and their regulatory interaction with Arabidopsis Cullin3-based E3 ligases. Plant cell.

[4] Stratmann JW, Gusmaroli G (2012). Many jobs for one good cop - the COP9 signalosome guards development and defense. Plant Sci..

[5] Dohmann EM, Kuhnle C, Schwechheimer C (2005). Loss of the CONSTITUTIVE PHOTOMORPHOGENIC9 signalosome subunit 5 is sufficient to cause the cop/det/fus mutant phenotype in Arabidopsis. Plant cell.

[6] Zhang W (2008). Genetic analysis of CAND1-CUL1 interactions in Arabidopsis supports a role for CAND1-mediated cycling of the SCFTIR1 complex. Proc. Natl. Acad. Sci. USA.

[7] Stuttmann J (2009). COP9 signalosome- and 26S proteasome-dependent regulation of SCFTIR1 accumulation in Arabidopsis. J. Biol. Chem..

[8] Huang H, Quint M, Gray WM (2013). The eta7/csn3-3 auxin response mutant of Arabidopsis defines a novel function for the CSN3 subunit of the COP9 signalosome. Plos one.

[9] Pacurar DI (2014). Identification of new adventitious rooting mutants amongst suppressors of the *Arabidopsis thaliana* superroot2 mutation. J. Exp. Bot..

[10] Delarue M, Prinsen E, Onckelen HV, Caboche M, Bellini C (1998). Sur2 mutations of *Arabidopsis thaliana* define a new locus involved in the control of auxin homeostasis. Plant J..

[11] Pacurar DI (2012). A collection of INDEL markers for map-based cloning in seven Arabidopsis accessions. J. Exp. Bot..

[12] Pacurar DI, Pacurar ML, Pacurar AM, Gutierrez L, Bellini C (2014). A novel viable allele of Arabidopsis CULLIN1 identified in a screen for superroot2 suppressors by next generation sequencing-assisted mapping. Plos one.

[13] Serino G (1999). Arabidopsis cop8 and fus4 mutations define the same gene that encodes subunit 4 of the COP9 signalosome. Plant cell.

[14] Lingaraju GM (2014). Crystal structure of the human COP9 signalosome. Nature.

[15] Cavadini S (2016). Cullin-RING ubiquitin E3 ligase regulation by the COP9 signalosome. Nature.

[16] Mosadeghi, R. *et al*. Structural and kinetic analysis of the COP9-Signalosome activation and the cullin-RING ubiquitin ligase deneddylation cycle. *eLife* **5**, doi:10.7554/eLife.12102 (2016). 10.7554/eLife.12102 PMC4878873 27031283

[17] Dohmann EM, Levesque MP, Isono E, Schmid M, Schwechheimer C (2008). Auxin responses in mutants of the Arabidopsis CONSTITUTIVE PHOTOMORPHOGENIC9 signalosome. Plant Phys..

[18] Wilkie AO (1994). The molecular basis of genetic dominance. J. Med. Genet..

[19] Stuttmann J, Parker JE, Noel LD (2009). Novel aspects of COP9 signalosome functions revealed through analysis of hypomorphic csn mutants. Plant Signal. Behav.

[20] Schwechheimer C (2001). Interactions of the COP9 signalosome with the E3 ubiquitin ligase SCFTIRI in mediating auxin response. Science (New York, N.Y.).

[21] Gutierrez L (2009). Phenotypic plasticity of adventitious rooting in Arabidopsis is controlled by complex regulation of AUXIN RESPONSE FACTOR transcripts and microRNA abundance. Plant cell.

[22] Gutierrez L (2012). Auxin Controls Arabidopsis Adventitious Root Initiation by Regulating Jasmonic Acid Homeostasis. Plant cell.

[23] Kwok SF, Piekos B, Misera S, Deng XW (1996). A complement of ten essential and pleiotropic arabidopsis COP/DET/FUS genes is necessary for repression of photomorphogenesis in darkness. Plant Phys..

[24] Franciosini A (2013). The Arabidopsis COP9 SIGNALOSOME INTERACTING F-BOX KELCH 1 protein forms an SCF ubiquitin ligase and regulates hypocotyl elongation. Mol. Plant.

[25] Wagner D, Hoecker U, Quail PH (1997). RED1 is necessary for phytochrome B-mediated red light-specific signal transduction in Arabidopsis. Plant cell.

[26] Wei N, Deng XW (1992). COP9: a new genetic locus involved in light-regulated development and gene expression in arabidopsis. Plant cell.

[27] Wei N, Chamovitz DA, Deng XW (1994). Arabidopsis Cop9 Is a Component of a Novel Signaling Complex Mediating Light Control of Development. Cell.

[28] Wei N (1998). The COP9 complex is conserved between plants and mammals and is related to the 26S proteasome regulatory complex. Curr. Biol..

[29] Chamovitz DA (1996). The COP9 complex, a novel multisubunit nuclear regulator involved in light control of a plant developmental switch. Cell.

[30] Sorin C (2005). Auxin and light control of adventitious rooting in Arabidopsis require ARGONAUTE1. Plant cell.

[31] Chen J (2013). Inhibition of arabidopsis hypocotyl elongation by jasmonates is enhanced under red light in phytochrome B dependent manner. J. Plant Res..

[32] Chico JM (2014). Repression of Jasmonate-Dependent Defenses by Shade Involves Differential Regulation of Protein Stability of MYC Transcription Factors and Their JAZ Repressors in Arabidopsis. Plant cell.

[33] Feng SH (2003). The COP9 signalosome interacts physically with SCFCOI1 and modulates jasmonate responses. Plant cell.

[34] Lozano-Duran R (2011). Geminiviruses subvert ubiquitination by altering CSN-mediated derubylation of SCF E3 ligase complexes and inhibit jasmonate signaling in *Arabidopsis thaliana*. Plant cell.

[35] Dohmann EM, Nill C, Schwechheimer C (2010). DELLA proteins restrain germination and elongation growth in *Arabidopsis thaliana* COP9 signalosome mutants. Eur. J. Cell Biol..

[36] Wang XP (2003). The COP9 signalosome interacts with SCFUFO and participates in Arabidopsis flower development. Plant cell.

[37] Chen H (2006). Arabidopsis CULLIN4 Forms an E3 Ubiquitin Ligase with RBX1 and the CDD Complex in Mediating Light Control of Development. Plant cell.

[38] Irigoyen ML (2014). Targeted degradation of abscisic acid receptors is mediated by the ubiquitin ligase substrate adaptor DDA1 in Arabidopsis. Plant cell.

[39] Dohmann EM (2008). The Arabidopsis COP9 signalosome is essential for G2 phase progression and genomic stability. Development.

[40] Grunewald W (2009). Expression of the Arabidopsis jasmonate signalling repressor JAZ1/TIFY10A is stimulated by auxin. EMBO reports.

[41] Hind SR (2011). The COP9 signalosome controls jasmonic acid synthesis and plant responses to herbivory and pathogens. Plant J.

[42] Salisbury FJ, Hall A, Grierson CS, Halliday KJ (2007). Phytochrome coordinates Arabidopsis shoot and root development. Plant J.

[43] Vitha S, Zhao L, Sack FD (2000). Interaction of root gravitropism and phototropism in Arabidopsis wild-type and starchless mutants. Plant Phys..

[44] Correll MJ (2003). Phytochromes play a role in phototropism and gravitropism in Arabidopsis roots. Advances in space research: the official journal of the Committee on Space Research.

[45] Laxmi A, Pan J, Morsy M, Chen R (2008). Light plays an essential role in intracellular distribution of auxin efflux carrier PIN2 in *Arabidopsis thaliana*. PloS one.

[46] Kircher S (1999). Light quality-dependent nuclear import of the plant photoreceptors phytochrome A and B. Plant cell.

[47] von Arnim AG, Deng XW (1994). Light inactivation of Arabidopsis photomorphogenic repressor COP1 involves a cell-specific regulation of its nucleocytoplasmic partitioning. Cell.

[48] Ang LH (1998). Molecular interaction between COP1 and HY5 defines a regulatory switch for light control of Arabidopsis development. Mol. Cell.

[49] Duek PD, Elmer MV, van Oosten VR, Fankhauser C (2004). The degradation of HFR1, a putative bHLH class transcription factor involved in light signaling, is regulated by phosphorylation and requires COP1. Curr. Biol..

[50] Seo HS (2003). LAF1 ubiquitination by COP1 controls photomorphogenesis and is stimulated by SPA1. Nature.

[51] Santoni V, Bellini C, Caboche M (1994). Use Of 2-Dimensional Protein-Pattern Analysis For The Characterization Of *Arabidopsis thaliana* Mutants. Planta.

[52] Abramoff MD, Magalhaes PJ, Ram SJ (2004). Image processing with ImageJ. Biophotonics International.

[53] Neff MM, Turk E, Kalishman M (2002). Web-based primer design for single nucleotide polymorphism analysis. Trends Genet..

[54] Rieu I, Powers SJ (2009). Real-time quantitative RT-PCR: design, calculations, and statistics. Plant cell.

[55] Andersen SU (2008). Requirement of B2-type cyclin-dependent kinases for meristem integrity in *Arabidopsis thaliana*. Plant cell.

