# Erratum: The Arabidopsis Cop9 signalosome subunit 4 (CSN4) is involved in adventitious root formation

**DOI:** 10.1038/s41598-017-04861-9

**Published:** 2017-07-20

**Authors:** Daniel Ioan Pacurar, Monica Lacramioara Pacurar, Abdellah Lakehal, Andrea Mariana Pacurar, Alok Ranjan, Catherine Bellini

**Affiliations:** 1 Umeå Plant Science Centre, Department of Plant Physiology, Umeå University, SE-90187 Umeå, Sweden; 2 University of Agricultural Sciences and Veterinary Medicine, 400372 Cluj Napoca, Romania; 3 Institut National de la Research Agronomic, UMR1318 INRA-AgroParisTech, Institut Jean-Pierre Bourgin, Univ. Paris-Sud, F-78000 Versailles, France; 4 SweTree Technologies AB, P.O. Box 4095, SE-904 03 Umeå, Sweden

*Scientific Reports* **7**:628; doi:10.1038/s41598-017-00744-1; Article published online 04 April 2017

In the original version of this Article, some instances of “CSN” were incorrectly given as “CNS”. This error has now been corrected in the PDF and HTML versions of the Article.

