# Supplementary material for: The Arabidopsis Cop9 signalosome subunit 4 (CSN4) is involved in adventitious root formation
Source: Sci Rep. 2017 Apr 4;7:628. doi: 10.1038/s41598-017-00744-1 (PMC5429640; doi:10.1038/s41598-017-00744-1)
Supplement: Supplementary file 1 — Supplementary information [file 41598_2017_744_MOESM1_ESM.pdf]

Supplementary Information

Title:

The Arabidopsis Cop9 signalosome subunit 4 (CNS4) is involved in adventitious root formation

Authors:

Daniel Ioan Pacurar*, Monica Lacramioara Pacurar, Abdellah Lakehal #, Andrea Mariana Pacurar #, Alok Ranjan and Catherine Bellini*

# These authors have equally contributed to the work

* Corresponding authors

Supplementary Figure S1

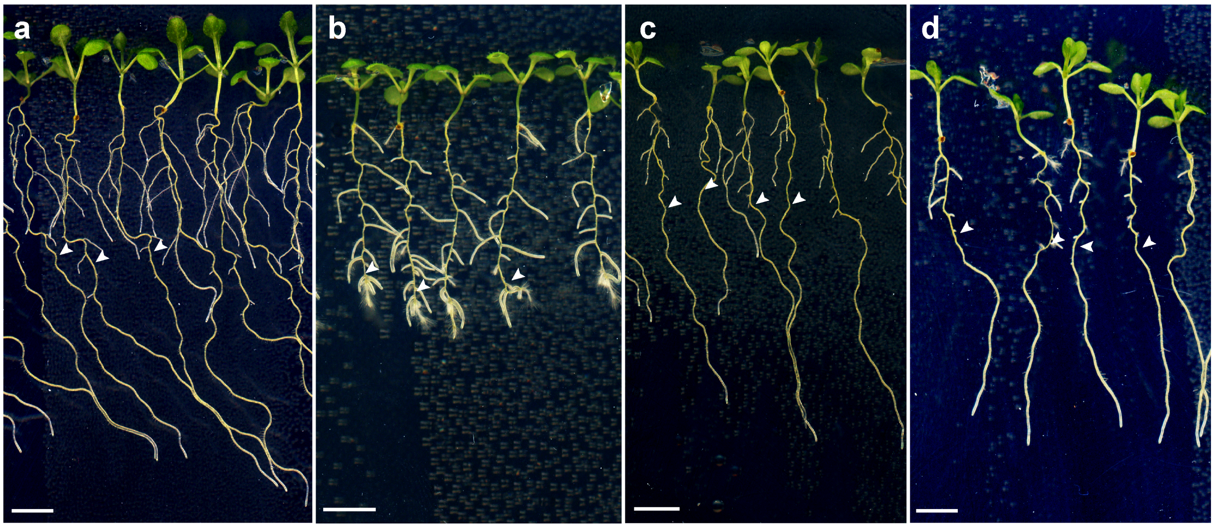

***csn4-2035* week-allele mutant is resistant to exogenous auxin.**

Wild-type Ws-4 seedlings (A, B) or *csn4-2035* (C, D) seedlings were grown in absence of 2,4-D for six days in long-day conditions then transferred to fresh medium in absence (A, C) or presence of 100 nM of 2,4-D and let grow for five additional days.

Arrow heads indicate the position of the primary root tip before transfer to fresh medium with or without 2,4-D.

Bar = 5 mm

Supplementary Figure S2

Experimental setup for hypocotyl length responsiveness of *Ws-4*, *sur2-1gl1* and *2035* to continuous red and blue light.

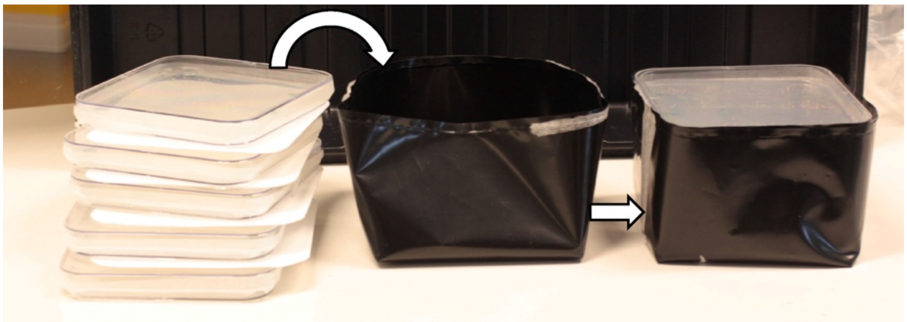

Supplementary Table S1.

Sequences of oligonucleotides used for genotyping the *csn4* alleles

Mutant	Primer name	Primer sequence	Source
csn4-1	CSN4-FW1	AAAAAGCGAGAGAGAAATCAAAACC	Dohmann E.M.N. et al., (2008)
	CSN4-RV1.1	CAATGCCACACACCTCATTC	This study
csn4-2	CSN4-FW2	TTTGCCTGACAAATCCACTG	Dohmann E.M.N. et al., (2008)
	CSN4-RV2	GCACAAGAAAGGTTCATCTATGC	Dohmann E.M.N. et al., (2008)
csn4-1 and csn4-2	LBb1.3	ATTTTGCCGATTTCGGAAC	http://signal.salk.edu/tdnaprimers.2.html
2035/ csn4-3	2035+HpaI.F	GAAGGTTATGCTCAATGTTA	This study
	2035+HpaI.R	GGCCACATCAGGTATGGACT	This study
csn1-10	csn1-10+Eco130I.F	CTTATCAACGATTTCTATCCAA	This study
	csn1-10+Eco130I.R	TAATTATGAGGGACTTCGTTGG	This study
csn2-5	Jo153/csn2.5+SalI.F	AGGAAGTCAGCTGCTTGAGG	Stuttmann J., personal communications
	Jo154/csn2.5+SalI.R	TGACGTTCTGCCATGTGCATTTGTCGA	Stuttmann J., personal communications
csn3-3	Ex7(csn3-3)+ Eco130I.F	CAACGACGGGAAGATTGGTG	Huang H. et al., (2013)
	Ex8(csn3-3)+ Eco130I.R	GCCTCCTTAGCATTACCAAG	Huang H. et al., (2013)

Supplementary Table S2.

Sequences of primers used for quantitative RT-PCR

Amplicon name	Gene	Forward primer	Reverse primer
ARF6 uncleaved	At1g30330	CAAAGTTTAGCAGCTACCACGA	ACGTCGTTCTCTCGGTCAAC
ARF8 uncleaved	At5g37020	TTTGCTATCGAAGGGTTGTTG	CATGGGTCATCACCAAGGA
ARF17 uncleaved	At1g77850	GCACCTGATCCAAGTCCTTC	GGTGAATAGCTGGGGAGGAT
GH3.3	At2g23170	ACAATTCCGCTCCACAGTTC	ACGAGTTCCTTGCTCTCCAA
GH3.5	At4g27260	GTCTTCGAGGACTGCTGCTT	ATGTCCCTGGCTCAACAATC
GH3.6	At5g54510	CCTTGTTCCGTTTGATGCTT	CGTGTTACCGTTCAAGCAGA
EF1A	At5g60390	TGGTGACGCTGGTATGGTTA	CCTTCTTGTCCACGCTCTT
